# Going to scale: dissemination and implementation of EBPs across large publicly-funded health care systems - initiatives targeting system-level change

**DOI:** 10.1186/1748-5908-10-S1-A31

**Published:** 2015-08-20

**Authors:** Stephen Crystal, Sheree Neese-Todd, Joel Cantor, Todd Gilmer

**Affiliations:** 1 Institute for Health, Health Care Policy, and Aging Research, Rutgers University, New Brunswick, NJ 08901, USA; 2 Center for Health Services Research on Pharmacotherapy, Chronic Disease Management and Outcomes/Center for Education and Research on Mental Health Therapeutics, Rutgers University, New Brunswick, NJ 08901, USA; 3 Center for State Health Policy, Rutgers University, New Brunswick, NJ 08901, USA; 4 Department of Family and Preventive Medicine, School of Medicine, University of California, San Diego, La Jolla, CA 92093, USA

## 

This panel will examine strategies and outcomes across three initiatives aimed at translating evidence-based care models and practices in the treatment of low-income, disadvantaged patients with behavioral health and other complex conditions at scale, across states and cities. The initiatives include translation across states of measurement-driven QI initiatives in mental health in the six-state MEDNET consortium; translation across cities of the care coordination model developed for low-income super-utilizers by Brenner and colleagues in Camden; and the implementation across 91 California sites of the Full Service Partnership (FSP) model for persons with severe mental illness who are homeless or at risk of homelessness.

The discussion will initially be framed by a brief overview from the organizer/discussant placing the three interventions within an overall conceptual model. Following the presentations, comments from the discussant will compare and contrast the effectiveness of the alternative strategies utilized across care processes and sites. Common themes across the three initiatives will be discussed, in terms of types of barriers and facilitators experienced in the process of dissemination at scale. We will discuss the barriers and facilitators experienced with respect to sustainability of the interventions beyond the initial project period; e.g., variations in successful incorporation metrics for evidence-based practices into ongoing management processes in public programs, and in incorporating guidelines for the use of evidence-based practices into state and health plan clinical guidelines, such as the Texas foster care psychotropic prescribing parameters and clinical guidelines promulgated by health plans across sites. We will discuss the important distinction between the types of barriers and facilitators experienced in the translation of specific program models across sites at small to medium scale, and the different set of barriers and facilitators that are encountered in efforts to bring these translational efforts to statewide and other large-scale systems with sustained impact.

Meaningful antipsychotic metrics for state medicaid programs: strategies for sustainable change and quality mental health care

Safe and effective use of antipsychotic medications across multiple populations, often off-label, presents many challenges for Medicaid programs. We will discuss the experience of a 6-state consortium, the Medicaid Mental Health Network for Evidence Based Care (MEDNET), in developing consensus quality metrics for safe and judicious antipsychotic use and translating measurement-driven QI initiatives at scale in large statewide populations. The presentation of barriers and facilitators experienced in development and use of metrics and translation of interventions across states with different policy and organizational environments.

## Data Sources

The state Medicaid and mental health systems in Texas, California, Missouri, Oklahoma, Maine and Washington.

Using mixed methods we assessed the uptake of metrics across states and the incorporation into sustainable, QI processes.

The metrics are feasible to implement, were spread to related agency projects, and integrated into ongoing QI efforts, with overall success but significant variation. Working with the National Collaborative for Innovation in Quality Measurement (NCINQ), several MEDNET metrics were adopted by NCQA as HEDIS 2015 measures. Significant impacts occurred in some settings: for example, a prior authorization requirement for antipsychotic prescriptions for children under age 3 in Texas led to a 57% decrease in the monthly number of very young children with such prescriptions and QI initiatives in six Washington community mental health centers were followed by 45% reduction in rate of polypharmacy. Factors associated with successful translation and sustainability included stable leadership, engagement of clinical champions, and willingness of state agencies to collaborate.

Translation across states requires creative adaptation of the models to differing policy and organizational environment. Identification of the factors associated with successful translation is key to translating effective practices at large scale.

Adapting the high utilization team model in four diverse sites

This project seeks to adapt an evidence-based care coordination program of the Camden Coalition of Healthcare Providers in four diverse clinical sites. The sites vary widely in delivery system and organizational characteristics - two health centers, one non-profit safety net medical center and one for-profit Independent Practice Association. One of the health centers has established a successful partnership with a behavioral health provider organization.

Setting: Allentown PA, Aurora CO, Kansas City MO, and San Diego CA.

Administrative and patient reported data are used to examine project implementation and fidelity to the Camden model. We hypothesize that the degree to which the Camden model is successfully adapted and the extent to which patient outcomes improve depends on organizational, delivery system, and policy context.

Achieving financial sustainability after three years of operations is a key project goal, but opportunities for sustainability will depend on the state policy context, which varies greatly across the sites. Data for the first 719 enrolled patients show that the majority are covered by Medicaid (including dual eligibles) or are uninsured, bear a high burden of chronic illness (e.g., 41% diabetes, 37% depression, and 21% COPD), and face substantial social challenges. The composition of enrollees (i.e., demographics, health status, and social needs such as housing), fidelity to the Camden model (e.g. intensity and duration of the intervention), and indicators of patient outcomes also vary widely across the sites. All sites evinced reductions in patient-reported unhealthy physical and mental health days and most decreased hospital utilization to varying degrees.

This project illustrates the challenges of program replication/adaptation as a dissemination strategy.

Variation in the implementation of California's full service partnerships for persons with serious mental illness

This study examined a large-scale implementation of supported housing programs under, California's Mental Health Services Act. Full Service Partnerships (FSPs), supported housing programs that do 'whatever it takes' to improve outcomes among persons with SMI who are homeless or at risk of homelessness evaluated. An emphasis on integrated, recovery-oriented care; flexible funding; and stakeholder influence led to the implementation of a diverse array of FSP programs. Data Sources: Ninety-three FSPs in California.

A mixed methods approach was selected to develop a better understanding of the complexity of the FSP programs. The design structure was a combined explanatory and exploratory sequential design where a focus group was used to develop a quantitative survey that was followed by site visits and analysis of administrative data. The survey was used to describe the breadth of variation based on fidelity to the Housing First model, while the site visits were used to provide a depth of information on high vs. low fidelity programs and the administrative data provided information on housing and services use.

Substantial variation in implementation exists among FSPs. Fidelity was particularly low along domains related to housing and service philosophy. Analysis of semi-structured interviews with program directors revealed fifteen themes organized into three domains of the Consolidated Framework for Implementation Research: individual characteristics of program directors, the inner setting and the outer setting. Interviews with front-line providers allowed us to identify an emergent model of recovery-oriented practice that integrated client choice with client agency. Additional analysis showed that higher fidelity programs enrolled clients with greater histories of homelessness who were less engaged in outpatient services.

There remains room for improvement in the fidelity and recovery-orientation of FSPs. We have identified several processes to increase fidelity.

